# Malignant Growths of the Skin: Their Diagnosis and Treatment

**Published:** 1894-06

**Authors:** M. B. Hutchins

**Affiliations:** Atlanta, Ga.; Clinical Lecturer on Dermatology and Syphilis in the Atlanta Medical College


### MALIGNANT GROWTHS OF THE SKIN: THEIR DIAGNOSIS AND TREATMENT.*

BY M. B. HUTCHINS, M. D.,
ATLANTA, GA.,
Clinical Lecturer on Dermatology and Syphilis in the Atlanta Medical College.

While not all the growths to be described are strictly malignant, in a literal sense, the term applies in that they are either malignant or terminate in malignancy.

We have the carcinomata of the skin, with epithelioma as a related disease, and the sarcomata.

Epithelioma being more frequently met with, will be described first.

Authors describe three varieties of epithelioma: The *superficial*, the *deep-seated*, the *papillary*.

The *superficial* usually begins as one or more pin-head to shot-sized, pale red, yellowish-white, grayish or dirty-looking, waxy, hard, firm, semi-transparent growths, which gradually form, from grouping, an irregular, wart-like, superficial mass, or a plate of infiltration, of various size and shape. Or it may begin in a wart, mole or other abnormal growth. The growth is at first movable with the skin, but it becomes fixed as it grows deeper. Soon there is fissuring or excoriation, starting from an accidental injury, from scratching, picking at it, or spontaneously. From these lesions there oozes a thin, scanty, sticky secretion which dries into a yellowish, or a dark, sero-sanguineous crust. At this stage it may simply resemble a wart which has been injured by scratching.

Certain little, round pearl-like bodies may be seen in the growth or at its borders, which are known as "cancroid corpuscles," or "epithelial pearls." They can easily be pressed out. The superficial form of epithelioma is at first of slow growth, but it is likely to enlarge and, sooner or later, break down and ulcerate. The ulcer is roundish or irregular, clean cut with sloping, raised, hard, firm, almost transparent edges. Its floor is reddish, uneven, granular, bleeds easily, it as well as the border, probably, showing dilated vessels. There is an oozing of a thin, scanty, sticky fluid

*Read before the late meeting of the Georgia Medical Association.

which dries into a varnish-like crust. It may remain a local sore for years or heal spontaneously, but it usually persists, sooner or later extending in breadth and depth. Robinson says that senile warts are liable to be the starting point of the superficial, and that it never involves the neighboring lymphatic glands.

The *deep-seated* variety may begin as the superficial, or start from a wart or a sebaceous tumor. It usually commences as roundish or conical, very firm, hard, translucent, reddish or purplish, pea-sized papules occupying the skin and subcutaneous tissue. There is usually, according to Shoemaker, an areola. It is generally elevated, but it may be flattened and diffuse, or it may, in months or years, grow to form a nut-sized, or larger, roundish, dark-reddish tumor, with fine, dilated vessels in its surface, and extensively infiltrating the tissues beneath and around it. Or it may take the form of a well-defined, thick, flattened patch, depressed in the center, with a shining, waxy or pinkish surface, showing dilated vessels. Other growths may develop in the vicinity, and the "pearls" may be seen at the borders.

Finally, ulceration; the ulcer resembling that of the superficial, or, being deeper, roundish or irregular, with clean cut, steep, everted, purplish, hard edges and a hard border around it. The base is granular, bleeds easily and shows a thick or thin, scanty, sticky, yellowish secretion, or the secretion may be purulent and foul. Sometimes its deep, immovable base shows an ulcer reaching to and attacking the bone. The glands become involved, break down, pain increases and death follows from this and exhaustion. Crocker says the deep-seated also begins in the subcutaneous tissue, with healthy skin over it, destruction then beginning deeply, forming at first only a small external opening.

The *papillary* form may at first resemble a wart, or it may begin on a wart, mole or other simple growth, or as a variously shaped smooth, spongy or raspberry-like growth, from half pea to nut size. The surface may be dry, reddish-yellow, shining, or it may be covered with a thin layer of horny, dried yellowish scales. The surface is moist and granular if it is in a moist situation. There may be a thick, sticky secretion mixed with blood, serum and a cheesy matter. If fissured, there is a similar secretion, perhaps offensive, forming brownish, adherent crusts, or there are abundant granulations. Ulceration finally. The ulcer is much like that of the other forms, roundish, uneven, the base and border hard, the edges undermined or everted, and of a purplish-red color. The granulations are small, it bleeds easily and exudes a thin, offensive fluid, which, if the disease is not in a moist situation, dries into a crust. Sooner or later there is a deep ulcer attacking every tissue in its course. The neighboring glands become cancerous, break down, the next in order may be affected and, eventually, even the lungs, liver and heart are involved by metastasis.

The sensations produced by epithelioma vary in different persons and in the different varieties, from simply a little discomfort to intense pain.

Epithelioma is a disease which chiefly affects those in or beyond middle-life. The face is its most common position, in the order of the eyelids and vicinity, the nose, lips, cheeks and forehead. It is also common on the penis, scrotum and labia. Delafield and Prudden say that it is more likely to occur at the junction of the skin and mucous membrane, and that metastasis is less likely to occur from epithelioma than from other malignant disease.

'The condition known as Paget's disease of the nipple may first appear as a simple inflammation, but it soon becomes a scirrhus cancer.'

The physician does not often see epithelioma in its early stages, but only after it has persisted long enough to induce the patient to think of having something done for it. We are, however, often able to judge as to the class to which it belongs.

The limits of this paper will not permit of my reporting cases from my records, but a number of them have already been published.

*Carcinoma of the Skin.*—Authors say that these are nearly always secondary to cancer of the breast or of some other organ. There are two varieties of this disease (omitting the melanotic, which is now considered a sarcoma), the *lenticular* and the *nodular.*

The *lenticular* begins as shot-sized, or larger, papules or nodules flattish, red, above or below the level, usually on a red or violet surface, but they may develop without color, and may show dilated vessels. Finally, from multiplication and coalescence, a thick, stiff, hard infiltration is formed, purplish or blackish in color, knobby and with a sharply defined border. This is the *carcinoma en cuirass* of Velpeau, occurring on the front of the chest. At the beginning there is only itching or burning; later there may be severe, lancinating pains. The neighboring glands become involved, and death finally results from pain and exhaustion, or from the disease in the internal organs.

The *nodular* form may be primary or secondary upon the skin. It is a rare form. The nodules are at first flat or raised, deep-set in the skin and subcutaneous tissues, well defined, rounded, hard and the skin covering them is often tense, and of any color from white to dark brown. The nodules are often numerous, grouped or scattered, or they may form irregular masses. Sooner or later there is softening, breaking down, ulceration, ending in death.

*Sarcoma of the Skin.*—Two kinds, the *pigmentary* and the *non-pigmentary*, are described. The pigmentary, or melanotic, is more common, according to the majority, but Duhring says there are more cases of the non-pigmentary than is usually supposed. Crocker describes two kinds of pigmentary, the melanotic and the the idiopathic multiple pigmented of Kaposi.

The *melanotic* may begin as one or more, at first small, discrete, roundish, hard growths, of brown, bluish or black color. It usually begins in a pigmentary mole or the choroid of the eye, but is common on the sides and back of the hands, fingers and feet, as well as the genitals. It rapidly invades every portion of the body. There is a gradual increase in size with coalescence into variously shaped masses which may later grow into a mushroom-like form and ulcerate. Some of the lesions, or of the mass, may undergo spontaneous atrophy, subsiding or giving the mass a depressed center. Entire absorption is followed by a pigmented scar. The pigmentary sarcoma is very malignant, metastasis being common. This form is usually primary in the skin.

The *idiopathic multiple, pigmented* form, is rare. It appears on the hands and feet as roundish, shot to pea-sized papules, reddish-brown or bluish in color, irregularly defined, fairly hard and in large or small groups. At the beginning the papules are separate, but they coalesce later. There is a gradual extension over the surface. The affected skin and subcutaneous tissues become densely infiltrated, stiffened, painful, hard and immovable. It has a nodular appearance, and is of a dark brown or a violet color. Crocker says that dilated vessels with hemorrhages around the tumors are common. It is not liable to ulceration. The average duration to death is three to five years, but shorter in young subjects. A few recoveries have been reported. The majority of cases occur in persons above middle age, but a number between the age of twenty and thirty, and one under twenty, have been reported. Delafield and Prudden say that sarcoma is a disease of youth.

The *non-pigmentary* form, the least malignant, may occur as one or many pea-sized to larger, round, firm, painless nodules of a reddish or pink color, and slightly scaly. They may arise from apparently healthy skin, from a mole, wart or scar. Crocker says these cases vary much, many being subcutaneous. Shoemaker says the growth may be rapid, and even reach orange size, eventually breaking down and ulcerating, and that it takes several years to involve the system. Other affections have been described which resemble the sarcomata in structure, but they are so rare as to excuse their non-mention here.

*The Treatment of Epithelioma.*—This must be limited to local measures, as internal treatment has no value. Incomplete destruction or removal is liable to do more harm than good. Whether caustics, the actual cautery, the galvano-cautery, ecraseur or knife are used, the operation must be thorough and the destruction of the growth complete. Duhring says the results are about equal where the methods are equally skilfully employed. The means used must be governed by the size, extent and situation of the growth. The caustics are said to be best for superficial growths. Caustic potash may be bored into the diseased tissue, and into its border, any excess being neutralized by dilute acetic acid. It is not an exceedingly painful method. Pure carbolic acid, followed in a few minutes by nitric acid, is mentioned by Shoemaker. Chloride of zinc has been used, but it is very painful, and has no advantages.

Marsden's Paste: Equal parts of arsenious acid and powdered gum acacia, mixed with sufficient water, is an excellent application for certain forms, as the small, not too deep. It is spread on a patch of cloth cut to fit the diseased area, and allowed to remain in place twenty-four hours. A little morphine may be added to lessen the pain. Cocaine's action is too transitory. The paste may be re-applied if it appears not to have had a complete effect. Poultices are used to hasten the separation of the eschar. A healthy wound will be left if all the disease has been destroyed, and the resulting scar is slight. Arsenic only attacks the disease, and this is a recommendation. The paste must not be applied to a surface of over two inches, and not at all to mucous membrane.

Bougard's Paste is as follows: Wheat flour, one dram; starch, one dram; arsenic, one grain; cinnabar, five grains; ammonium chloride, five grains; mercuric chloride, one-half grain; saturated solution of chloride of zinc, four drams and five grains. The solids are ground together into a fine powder and mixed in a mortar; then while it is rapidly stirred the zinc solution is slowly added. As much as is necessary to cover the disease is allowed to remain on from twenty-four to thirty hours, and followed by poultices. Its action seems all that could be desired. Various other pastes and applications have been recommended, but they have no especial excellence. The object is to destroy the growth and leave a healthy wound. If any disease remains, the wound will not heal kindly, and treatment must be repeated. Sometimes the wound heals, but soon breaks down again with evidences of the disease. Recurrences may finally be destroyed and a cure accomplished. The various cauteries have their use sometimes, that use being governed by circumstances.

The curette may be used for very superficial growths, or preceding the caustic application.

The knife is the last to be mentioned, but it should be first in use. There is a popular prejudice against the knife, and people will submit to anything in preference to its employment. There are recurrences after the knife; but their frequency, their malignancy and their terribly destructive action following inefficient or incompetently used *applications* remove none of the fear of "the knife." We have all seen moving arguments against the temporizing, or worse, methods so often employed, and the people have seen them, but without learning the lesson taught. For the majority of cases the knife is the only remedy, and it is to be preferred where not mechanically contra-indicated.

The deep-seated and the papular especially require excision Every visible particle of the disease must be removed, and with it a perfect coating of healthy tissue. If neighboring glands are involved, they must be removed, and they may contain the disease without any visible evidence of the fact. Whether the excision wound shall be treated as an open one, by grafting, transplantation or by a plastic operation, must depend upon the case and the size of the wound. There are recurrences after all methods of treatment, sometimes curable, sometimes not. Taken early and while yet a local or limited disease, and thoroughly removed or destroyed, the result should be perfect cure. The trouble is that we can often only decide with the microscope whether we have removed all.

There is little to be said regarding the treatment of the cutaneous carcinomata and sarcomata.

The carcinoma is so likely to be secondary that the local treatment is of doubtful use. We must direct our attention to general palliative and supporting measures.

If the sarcomatous growths are few or single, excision may answer. Fowler's solution of arsenic, in gradually increased doses, has been reported as having cured a few cases. It is worthy of trial.

Persons suffering with a hopeless malignant disease usually resort to morphine, and why should we try to restrict their use of it? If their pain and wretchedness can be mitigated, why not allow it, and make the downward journey as easy as possible?

Authorities consulted: Robinson, Duhring, Shoemaker, Crocker, Hyde Delafield and Prudden.

